# Discoidin domain receptor 1 activity drives an aggressive phenotype in gastric carcinoma

**DOI:** 10.1186/s12885-017-3051-9

**Published:** 2017-01-31

**Authors:** Hoon Hur, In-Hye Ham, Dakeun Lee, Hyejin Jin, Kristina Y. Aguilera, Hye Jeong Oh, Sang-Uk Han, Ji Eun Kwon, Young-Bae Kim, Ke Ding, Rolf A. Brekken

**Affiliations:** 1 Division of Gastrointestinal Surgery, Department of Surgery, Ajou University School of Medicine, 164 Worldcup-ro, Yeongtong-gu, Suwon 443-380 South Korea; 2 Brain Korea 21 Plus Research Center for Biomedical Sciences, Ajou University, Suwon, Korea; 3 Department of Pathology, Ajou University School of Medicine, Suwon, Korea; 4 State Key Laboratory of Respiratory Diseases, Guangzhou Institutes of Biomedicine and Health, Chinese Academy of Sciences, Guangzhou, China; 5 Division of Surgical Oncology, Department of Surgery, Hamon Center for Therapeutic Oncology Research, University of Texas Southwestern Medical Center, 6000 Harry Hines Boulevard, Dallas, TX 75390-8593 USA

**Keywords:** Cancer stroma, Discoidin domain receptor 1, Gastric carcinoma, Prognosis

## Abstract

**Background:**

Discoidin domain receptor 1 (DDR1), a receptor tyrosine kinase that utilizes collagen as a ligand, is a key molecule in the progression of solid tumors as it regulates the interaction of cancer cells with the tumor stroma. However, the clinical relevance of DDR1 expression in gastric carcinoma is yet to be investigated. Here, we assessed the role of DDR1 in mediating the aggressive phenotype of gastric carcinoma and its potential as a therapeutic target.

**Methods:**

We conducted DDR1 immunohistochemistry using a tissue microarray of 202 gastric carcinoma specimens. We examined the effect of collagen-induced activation of DDR1 on cell signaling, tumorigenesis, and cell migration in gastric cancer cell lines, and tumor growth in a xenograft animal model of gastric cancer.

**Results:**

Our results showed that 50.5% of gastric cancer tissues are positive for DDR1 expression, and positive DDR1 expression was significantly correlated with a poor prognosis (*P* = 0.015). In a subgroup analysis, DDR1 expression was prognostically meaningful only in patients receiving adjuvant treatment (*P* = 0.013). We also demonstrated that collagen was able to activate DDR1 and increase the clonogenicity and migration of gastric cancer cells. We observed that a DDR1 inhibitor, 7rh benzamide, suppressed tumor growth in gastric cancer xenografts.

**Conclusions:**

Our findings suggest a key role for DDR1 signaling in mediating the aggressive phenotype of gastric carcinoma. Importantly, inhibition of DDR1 is an attractive strategy for gastric carcinoma therapy.

**Electronic supplementary material:**

The online version of this article (doi:10.1186/s12885-017-3051-9) contains supplementary material, which is available to authorized users.

## Background

Gastric cancer is among the most common malignancies and the third leading cause of cancer-related death worldwide [1]. The gastric cancer mortality rate has slightly decreased due to an increase in curative surgical resection. However, recurrence is common in most patients with late-stage disease [2]. Recent large-scale clinical trials have demonstrated the efficacy of 5-fluorouracil-based adjuvant chemotherapy to reduce recurrence following curative resection in the patients with stage II or III cancer [3, 4]. However, recurrence is still diagnosed in approximately 25% of these patients despite the use of adjuvant treatments. Further, conventional chemotherapy is not effective in these patients and in those with initially unresectable disease [5–7]. Targeted therapies directed against cancer-specific molecules have been shown to increase survival in several solid-tumor cancers [8, 9]. However, the success of these agents has been modest in gastric cancer patients [10]. For example, two clinical studies showed limited clinical benefits from trastuzumab and ramucirumab for gastric cancer treatment [11, 12]. Thus, there is a pressing need for novel therapeutic agents to suppress the recurrence and progression of gastric cancers and a need for new biomarkers to predict tumor recurrence.

Discoidin domain receptors (DDRs) are members of the transmembrane receptor tyrosine kinases (RTKs) and uniquely possess a discoidin homology domain in their extracellular region [13–15]. Distinct from other RTKs, which are typically activated by growth factor ligands, DDRs use various types of triple-helix collagens, a main component of the extracellular matrix, independently of the integrin collagen receptors. To date, two DDRs, DDR1 and DDR2, have been identified. These DDRs display minor differences in their ligand specificities. DDR1 is activated by most collagens such as the type I, II, III, IV, V, VIII, and XI collagen. In contrast, DDR2 uses only collagen type I and III as its ligands [15–17]. Various studies have suggested that a collagen-activated DDR1 signaling pathway can enhance the self-renewal, spreading, migration, and tubulogenesis of non-cancerous cells [18–21]. These findings have encouraged further studies to elucidate the role of DDR1 in cancers. DDR1 expression is higher in solid malignant tumors than in normal tissues [22], and elevated DDR1 expression has been associated with a poor prognosis in pancreatic and lung cancers [23, 24]. However, the significance of DDR1 expression in gastric cancers is yet to be evaluated.

Gastric cancers are known to progress through the interaction between cancer cells and the tumor stroma [25, 26]. Therefore, the elevated expression of DDR1, which uses the stromal collagen as a ligand, might be a novel target for gastric cancer treatment. Moreover, novel agent, the 7rh compound of 3-(−2-(pyrazolo[1,5-a] pyrimidin-6-yl)-ethynyl) benzamides, to inhibit DDR1 activation was introduced recently. The preclinical study showed that it significantly suppressed the proliferation of DDR1-expressing cells [27]. Here, we investigated the clinical correlation of DDR1 expression in gastric cancers and determine if a novel DDR1-targeting agent, 7rh benzamide, can suppress cancer progression.

## Methods

### Patients and tissue samples

Of the patients diagnosed with gastric carcinoma and undergone curative gastric resections with proper lymphadenectomy at the Department of Surgery of Ajou University Hospital from May 2003 to December 2005, we selected 202 patients with the paraffin-embedded tissues which were possible to make a tissue microarray (TMA). All patients in the study were treatment-naïve. The median follow-up period for these patients was 67.4 ± 18.1 (median ± standard deviation) months. Recurrence was assessed with computed tomography of the abdominal and pelvic cavities, gastroscopy, or tumor markers every 3 or 6 months. Pathology stages were re-evaluated according to the seventh edition of the International Union Against Cancer classification criteria. Of histological subtypes determined according to the World Health Organization classification, papillary and well- or moderately differentiated tubular adenocarcinomas were classified as differentiated tumors, while other types were classified as undifferentiated tumors. Patients diagnosed with stage II or III disease were recommended for adjuvant systemic chemotherapy with 5-fluorouracil for 6 months or 1 year post surgery.

Two experienced pathologists (Kim YB and Kwon JE) reviewed all hematoxylin and eosin (H&E)-stained slides to designate appropriate sites for TMA cores. Two formalin-fixed and paraffin-embedded cores (2 mm in diameter) were removed from the selected sites and arranged into the TMA block.

### Immunohistochemistry

Immunohistochemistry (IHC) analyses were performed with 4-μm-thick section slides from the TMA block. Following antigen retrieval with citrate buffer and inhibition of endogenous peroxidase activity, the slides were incubated with DDR1 antibody (1:100 dilution, #sc-532, Santa Cruz Biotechnology, Dallas, TX) overnight at 4 °C. Reactivity was visualized after incubation with HRP-conjugated anti-rabbit secondary antibody (1:500 dilution, abC-5003, AbClon, Seoul, Korea) and the addition of the 3,3-diaminobenzidine substrate. The specificity of the anti-DDR1 antibody was assessed by Western blot analysis using the MKN45 gastric cancer cell line transfected with control or DDR1 siRNA (Additional file 1: Figure S1).

Stained slides were independently evaluated by two pathologists who were blinded to the clinicopathological features of the patients. Proportion of cells with positive DDR1 staining was determined from 0 to 100%, and the results were semi-quantitatively scored as follows: negative staining (<5%), 1+ staining (5–30%), 2+ staining (30–60%), and 3+ staining (>60%). The expression of DDR1 was determined as positive when both sites received a 2+ or 3+ score.

### Cell lines and chemical compounds

Gastric cancer cell lines KATO-III (KCLB No. 30103) and MKN28 (KCLB No. 80102) were purchased from the Korean Cell Line Bank (Seoul, Korea). The cells were maintained in RPMI-1640 (Invitrogen, Carlsbad, CA) containing 10% fetal bovine serum (FBS; Equitech-Bio, Ingram, TX) supplemented with 100 U/ml penicillin G and 100 μg/ml streptomycin (Invitrogen). Cells were incubated at 37 °C in a humidified atmosphere containing 20% O_2_ and 5% CO_2_. Type I collagen extracted from rat tail (BD Biosciences, Franklin Lakes, NJ) was dissolved in 0.02 N acetic acid and used for coating tissue culture dishes and inserts for migration assays (5 μg/cm^2^). Collagen-coated dishes and inserts were washed with PBS immediately before use.

DDR1 inhibitor 3-(2-(pyrazolo[1,5-a]pyrimidin-6-yl)ethynyl)benzamide (7rh benzamide) was dissolved in DMSO to a final stock concentration of 2.5 mg/ml as previously reported [27].

### Western blotting

Sub-confluent monolayers of cells were lysed and supernatants were recovered by centrifugation. Protein concentrations were measured using the BCA protein assay kit (Thermo Scientific, Waltham, MA) and equal amounts of total protein were resolved using SDS-PAGE gels. Resolved proteins were transferred to polyvinylidene difluoride membranes (Bio-Rad, Hercules, CA) and blocked with 5% milk in TBS/Tween-20 (TBST). Membranes were incubated with the following primary antibodies: DDR1 (1:1,000 dilution, #5583, Cell Signaling Technology, Danvers, MA), phosphorylated-DDR1 (1:1,000 dilution, #11994, Cell Signaling Technology), phosphorylated-PYK2 (1:1,000 dilution, #3291, Cell Signaling Technology), E-cadherin (1:2,000, 610181, BD Biosciences), and beta-actin (1:5,000 dilution, sc-47778, Santa Cruz Biotechnology), followed by the corresponding HRP-conjugated secondary antibodies (Jackson ImmunoResearch Labs, West Grove, PA). Protein bands were detected using the enhanced chemiluminescence reagent kit (Thermo Scientific) on autoradiographic films.

### Clonogenic assay

Cells were cultured in 6-well tissue culture dishes with or without collagen coating at 3 × 10^3^ or 5 × 10^3^ cells/well density in 2 ml RPMI-1640 supplemented with 5% FBS. Colony formation was visualized with crystal violet staining after fixation with 6% glutaraldehyde. Images were captured using a dissection microscope and analyzed with ImageJ software (US National Institutes of Health, Bethesda, MD) to assess colony size and numbers.

### Migration assay

Cell migration assays were performed using a two-chambers transwell cell culture system with 8 μm pore polycarbonate membrane inserts (3422, Corning, Cambridge, MA). To evaluate the effect of collagen on the migration ability of cancer cells, the upper side of the transwell membrane was coated with rat tail collagen. Cells were seeded at a density of 2 × 10^4^ cells/200 μl onto the upper chamber of the transwell in FBS-free medium. Cells were then allowed to migrate toward the lower chamber, which contained medium with 10% FBS for 48 hours at 37 °C in a humidified incubator. Migrated cells were fixed with 70% methanol and stained with H&E. Stained cells were visualized and photographed under an inverted bright-field microscope at 100× magnification, and counted using ImageJ software.

### Cell viability test

Cells were seeded at 1 × 10^5^ cells per well in 96-well plates, and cell viability was daily measured using the novel tetrazolium compound 3-(4,5-dimethylthiazol-2-yl)-5-(3-carboxymethoxyphenyl)-2-(4-sulfophenyl)-2H-tetrazolium inner salt [MTS(s)] assay kit (Promega, Madison, WI, USA). Next, 20 μl of the MTS solution was added per well, cells were incubated for 2 h, and the absorbance was measured by spectrophotometry at 490 nm. To evaluate the effect of 7rh benzamide on the cell viability, the cells were treated with different concentrations during 72 h. Three independent experiments were performed.

### In vivo assay

Six- to eight-week-old male *BALB/c-nu* nude mice (Orient Bio, Gyonggi-Do, Korea) weighing 16 to 18 g were subcutaneously implanted with 1 × 10^7^ MKN28 cells in 50 μl volume. We performed two experiments. Experiment 1: Mice (*n* = 10) were observed after MKN28 injection. Five mice were sacrificed on day 5 and 12 mice were sacrificed post injection, and tumors were harvested for histological evaluation. Experiment 2: Mice were injected subcutaneously with MKN28 cells. On day 1 after the tumor cell injection, mice were treated orally with 7rh benzamide (25 mg/kg, *n* = 6) or vehicle (*n* = 8) every 2 days for 15 days. Tumor volume and body weight were monitored throughout the study period.

In all experiments, tumors were measured in three dimensions using calipers, and tumor volume was calculated with this formula: tumor volume (mm^3^) = (*a* x *b* ^2^)/2, where *a* = length in mm, and *b* = width in mm.

Upon sacrifice, tumors were harvested, fixed in 10% neutral-buffered formalin, embedded in paraffin, and sectioned for staining as described for the human TMA study. Additional immunofluorescence stains were performed with antibodies against: phosphorylated-DDR1 (Tyr792; 1:100 dilution, #11994, Cell Signaling Technology), phosphorylated-PYK2 (Tyr402; 1:100 dilution, #3291, Cell Signaling Technology), and E-cadherin (1:50 dilution, 610181, BD Biosciences). Fluorescent images were obtained using an Olympus ix71 microscope equipped with a DP70 camera and DP controller software (Olympus, Tokyo, Japan). Fluorescent intensities were assessed using ImageJ software.

### Statistical analysis

All in vitro studies were performed three times independently. Statistical analysis was performed using the IBM SPSS statistics (Version 21 for Mac OS X, IBM, Armonk, NY) and GraphPad Prism (Version 6.0 for Mac OS X, GraphPad Inc., La Jolla, CA) software. Correlations between the expression of each molecule and the clinicopathological factors were evaluated using the χ^2^ test. Overall survival rates were evaluated using log-rank tests, and survival curves were generated using the Kaplan-Meier method. Mean value differences between two groups with continuous variables were evaluated using an independent t-test or Mann-Whitney U test.

## Results

### DDR1 expression in gastric normal and cancer tissues

**Fig. 1:**
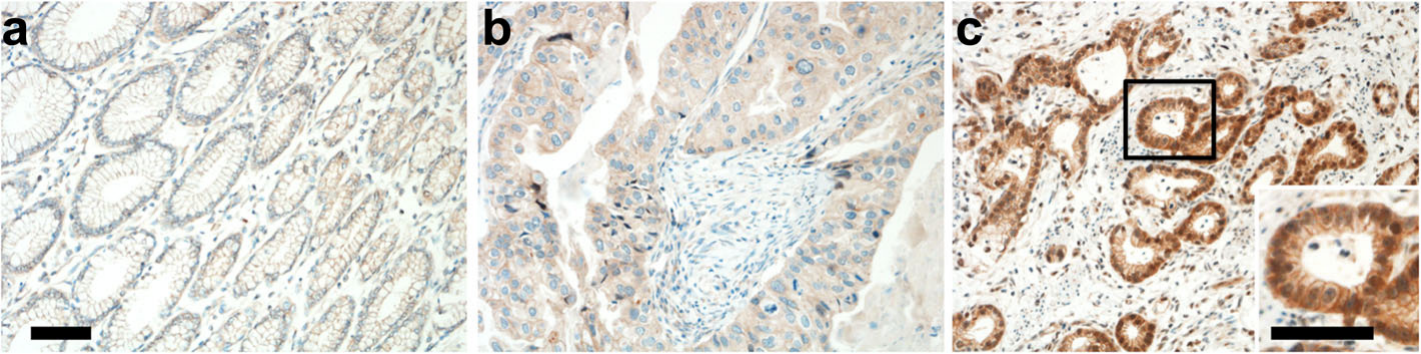
DDR1 expression in normal gastric and cancer tissues of patients with gastric cancer. Representative images of **a**) negative DDR1 immunohistochemical staining of normal gastric tissue, **b**) negative DDR1 staining in gastric cancer tissue with a +1 score, and **c**) positive DDR1 staining in gastric cancer tissue with a +3 score. The Inset shows the magnified positive DDR1 staining cancer cells. Scale bar = 200 μm

DDR1 was not expressed in normal gastric tissues (Fig. 1a). DDR1 staining was generally observed at the cell membrane or in the cytoplasm of tumor cells, and 50.5% of gastric cancer tissues showed positive DDR1 expression (Fig. 1b and c). Positive expression of DDR1 was associated with tumor invasion (*P* = 0.017). DDR1 positive staining was observed in 41.2% of T1 and T2 primary tumors and in 58.8% of T3 and T4 tumors. Other clinicopathological features were not correlated with DDR1 expression (Table 1).

**Table 1:** DDR1 expression in gastric cancer tissues according to various clinicopathological features

			DDR1		*P* value
Variables			Negative	Positive	
			(*n*=100)	(*n* = 102)	
Age (years old)	<70	147	70 (47.6%)	77 (52.4%)	0.381
	≥70	55	30 (54.5%)	25 (45.5%)	
Gender	Male	140	72 (51.4%)	68 (48.6%)	0.411
	Female	62	28 (45.2%)	34 (54.8%)	
Location	Upper	26	12 (46.2%)	14 (53.8%)	0.597
	Middle	62	34 (54.8%)	28 (45.2%)	
	Low	114	54 (47.4%)	60 (52.6%)	
Lauren	Intestinal	99	44 (44.4%)	55 (55.6%)	0.085
	Mixed	33	22 (66.7%)	11 (33.3%)	
	Diffuse	70	34 (48.6%)	36 (51.4%)	
Differentiation	Differentiated	70	35 (50.0%)	35 (50.0%)	0.918
	Undifferentiated	132	65 (49.2%)	67 (50.8%)	
T stage	T1/T2	100	58 (58.0%)	42 (42.0%)	0.017
	T3/T4	102	42 (41.2%)	60 (58.8%)	
N stage	N0/N1	125	66 (52.8%)	59 (47.2%)	0.233
	N2/N3	77	34 (44.2%)	43 (55.8%)	
Adjuvant chemotherapy	None	59	32 (54.2%)	27 (45.8%)	0.388
	Yes	143	68 (47.6%)	75 (52.4%)	

**Fig. 2:**
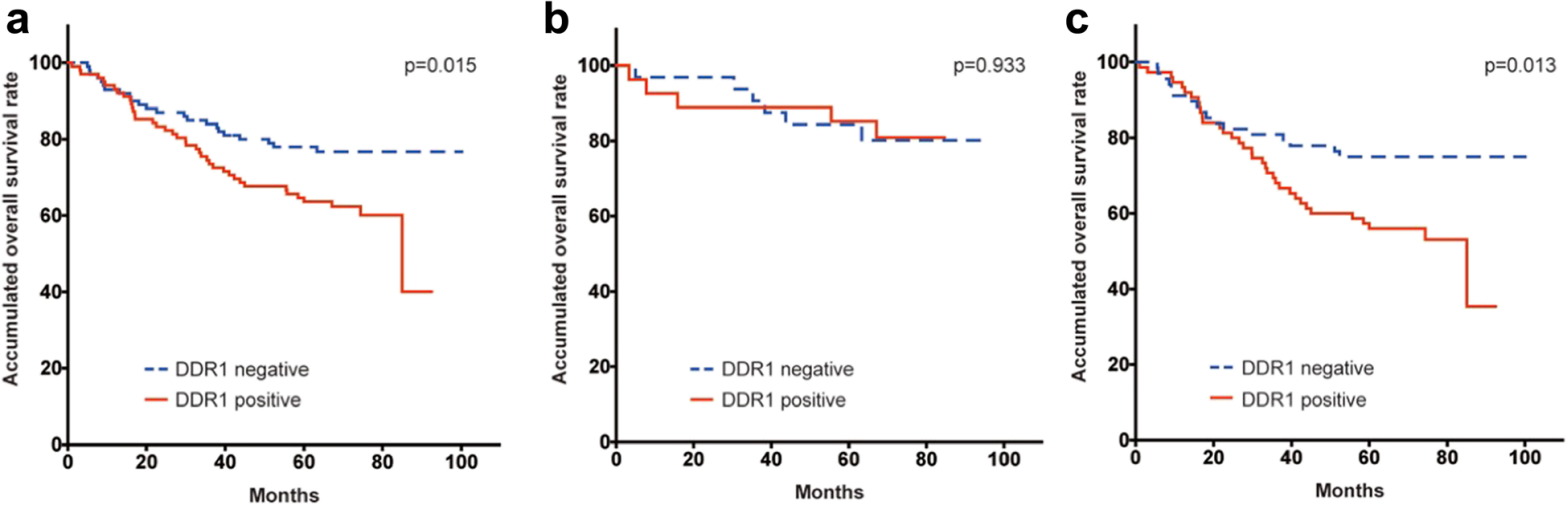
Overall survival of gastric cancer patients with positive or negative DDR1 expression. **a** Kaplan-Meier survival curves showed that positive DDR1 expression was associated with a lower overall survival rate in all patients enrolled in the study (*P* = 0.015 by log-rank test). **b** In the non-adjuvant chemotherapy subgroup, the overall survival rate of patients with positive and negative DDR1 expression was comparable (*P* = 0.933 by log-rank test). **c** In patients receiving adjuvant chemotherapy, positive DDR1 expression was significantly correlated with a poor overall survival rate (*P* = 0.013 by log-rank test)

Patients with positive DDR1 expression showed a worse prognosis when compared to DDR1-negative patients (*P* = 0.015; Fig. 2a). Old age, as well as advanced T and N stages were also associated with a poor prognosis. In a multivariate analysis, DDR1 expression was not a predictor of poor overall survival; however, old age, being male, having an undifferentiated tumor, and advanced T stage were all significant factors of poor survival (Table 2). We subsequently classified patients based on their adjuvant chemotherapy status. In the non-adjuvant treatment subgroup, DDR1 expression was not associated with a worse overall survival rate (*P* = 0.933; Fig. 2b). However, in patients receiving adjuvant treatment, DDR1 expression was significantly correlated with poor survival (*P* = 0.013; Fig. 2c). DDR1 expression was an independent prognostic factor in the multivariate analysis (*P* = 0.083, odds ratio = 1.7; Table 2).

**Table 2:** Univariate and multivariate analyses of clinicopathological features and overall survival rate of patients enrolled in the study

		Univariate analysis						Multivariate analysis					
		Total		No adjuvant		Adjuvant		Total		No adjuvant		Adjuvant	
		Mean ± SD	*P* value	Mean ± SD	*P* value	Mean ± SD	*P* value	OR (95% CI)	*P* value	OR (95% CI)	*P* value	OR (95% CI)	*P* value
Age	<70	80.0 ± 2.8	0.019	82.5 ± 3.6	0.028	76.5 ± 3.4	0.016	1	<0.001			1	0.002
	≥70	67.2 ± 4.7		78.2 ± 6.1		52.0 ± 5.2		2.6 (1.6–4.4)				2.5 (1.4–4.5)	
Gender	Male	74.1 ± 3.1	0.092	79.7 ± 4.6	0.027	70.6 ± 3.9	0.346	1.8 (1.0–3.3)	0.048				
	Female	75.7 ± 3.7		NR		70.5 ± 4.8							
Location	Upper	78.8 ± 7.1	0.109	66.1 ± 11.4	0.961	77.2 ± 8.1	0.130						
	Middle	81.8 ± 3.5		86.3 ± 4.7		76.2 ± 4.5							
	Low	80.0 ± 2.5		78.1 ± 4.5		61.3 ± 3.5							
Lauren	Intestinal	78.0 ± 3.6	0.651	86.9 ± 2.5	0.374	63.7 ± 4.1	0.644						
	Mixed	73.6 ± 5.0		79.7 ± 5.0		75.1 ± 4.7							
	Diffuse	68.1 ± 3.6		76.2 ± 2.4		68.9 ± 6.4							
Differentiation	Differentiated	84.0 ± 3.7	0.064	83.4 ± 4.9	0.571	82.9 ± 4.9	0.042	1	0.042			1	0.072
	Undifferentiated	68.8 ± 2.9		79.3 ± 4.3		64.7 ± 3.4		1.8 (1.0–3.3)				1.9 (0.9–3.8)	
T stage	T1/T2	90.3 ± 1.7	<0.001	91.6 ± 2.0	<0.001	83.9 ± 2.1	<0.001	1	<0.001	1	0.006	1	<0.001
	T3/T4	60.7 ± 4.0		41.8 ± 10.3		62.0 ± 4.2		6.4 (3.8–12.1)		7.0 (1.7–28.2)		4.8 (2.1–10.8)	
N stage	N0/N1	82.9 ± 2.5	<0.001	87.6 ± 3.2	0.003	75.3 ± 3.1	0.005			1	0.039		
	N2/N3	62.8 ± 4.5		54.7 ± 11.0		63.1 ± 4.8				4.8 (1.1–21.5)			
DDR1	Negative	82.8 ± 3.3	0.015	84.7 ± 4.3	0.933	80.4 ± 4.2	0.013					1	0.083
	Positive	66.5 ± 3.6		74.5 ± 4.6		62.2 ± 3.9						1.7 (0.9–3.0)	

These results indicated the potential of DDR1 expression as a new biomarker to predict poor survival of gastric cancer patients. In particular, its clinical relevance was more significant for patients receiving adjuvant treatment after resection.

### DDR1 signal transduction and its inhibition in gastric cancer cell lines

**Fig. 3:**
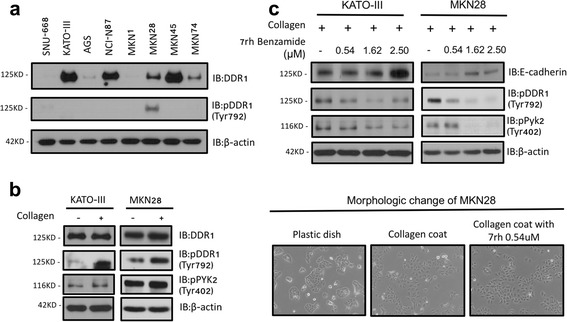
Collagen-induced activation and pharmacologic inhibition of DDR1 in vitro. **a** DDR1 and phosphorylated DDR1 levels in various gastric cancer cell lines were determined by Western blotting. **b** KATO-III and MKN28 cells were stimulated with rat tail type I collagen. Lysates were probed for indicated proteins by Western blotting. **c** KATO-III and MKN28 cells plated on collagen-coated dishes were treated with 7rh benzamide (0.18, 0.54, and 1.62 μM). The level of phosphorylated DDR1 and PYK2, E-cadherin, and β-actin were determined by Western blotting. **d** MKN28 cell morphology was changed into a more linear and mesenchymal-like shape by collagen stimulation, and this altered morphology was partially inhibited by 7rh benzamide treatment. The representative images are shown

To extend these findings, we investigated the expression of DDR1 in various human gastric cancer cell lines (Fig. 3a). In KATO-III and MKN28 cell lines, which highly express DDR1 compared to SNU-668 and AGS, collagen stimulation induced the phosphorylation of DDR1 and PYK2 (Fig. 3b). In both cell lines, the inhibition of DDR1 activity with 7rh benzamide decreased the phosphorylation of DDR1 and PYK2 in a dose-dependent manner. E-cadherin expression was higher in both cell lines (Fig. 3c). Furthermore, collagen stimulation altered the morphology of cells into a more linear and mesenchymal-like shape, and this altered morphology was partially inhibited by 7rh benzamide treatment (Fig. 3d). These results showed that collagen could activate DDR1 and its downstream signaling, and induce an epithelial-mesenchymal transition (EMT) via the loss of E-cadherin in gastric cancer cell lines. In addition, the ability of 7rh benzamide to inhibit the DDR1 signaling pathway was demonstrated.

### Collagen-DDR1 signaling promoted tumor aggressiveness

Cell viability tests for KATO-III and MKN28 cell lines did not show the difference between collagen coating dish and non-coating dish during 4 or 6 days (Additional file 2: Figure S2A and S2B). Moreover, low dose of 7rh benzamide (1 μM or less) did not give an effect on the cell viability in collagen coating and non-coating dishes (Additional file 2: Figure S2C and S2D).

**Fig. 4:**
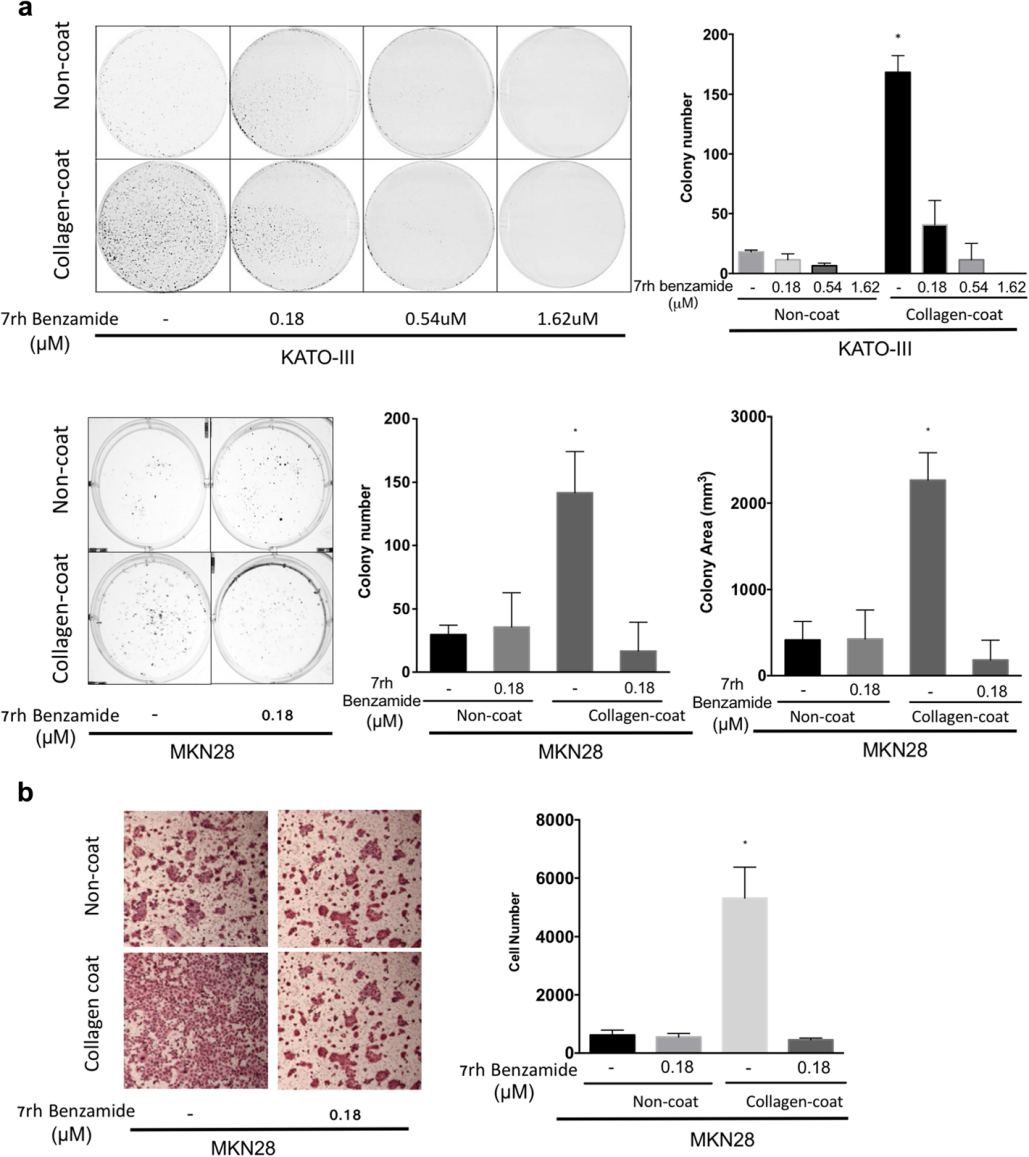
Colony formation and migration of gastric cancer cells were stimulated by collagen and reduced by DDR1 inhibition. **a** KATO-III and MKN28 cells (3 × 10^3^) were plated on plastic or collagen-coated dishes for 7 days and the effect of 7rh benzamide (0.18, 0.54, and 1.62 μM) on colony formation was determined. The number and total area of colonies were assessed using ImageJ software. **b** The effect of DDR1 inhibition on MKN28 cell migration was determined in a transwell migration assay. Transwell inserts (8 μm pore) were left uncoated or coated with collagen, and cells (3 × 10^4^) were seeded in serum-free medium. Complete medium containing 10% FBS was placed in the lower chambers, and cells were allowed to migrate for 2 days. The underside of each membrane was stained with H&E, and cells were counted using ImageJ software. The error bars represent standard error of the mean (SEM) and an asterisk (*) represents *P* < 0.005, calculated by one-way ANOVA with Tukey’s multiple comparison test

The overall capacity of gastric cancer cells to form colonies was significantly enhanced in the presence of collagen, specifically the number of colonies formed and the area covered by colonies. Moreover, 7rh benzamide (0.18 μM) was effective in reducing colony formation as well as colony size in KATO-III and MKN28 cell lines (Fig. 4a).

We subsequently assessed the contribution of collagen in the enhancement of cell motility using a transwell migration assay. We observed that collagen increased cell migration, while 7rh benzamide treatment decreased cell motility (Fig. 4b). These results demonstrated that the inhibition of DDR1 by 7rh benzamide decreased the tumorigenesis and migration of gastric cancer cell lines.

### The inhibition of DDR1 reduced gastric tumor growth following stromal collagen accumulation

**Fig. 5:**
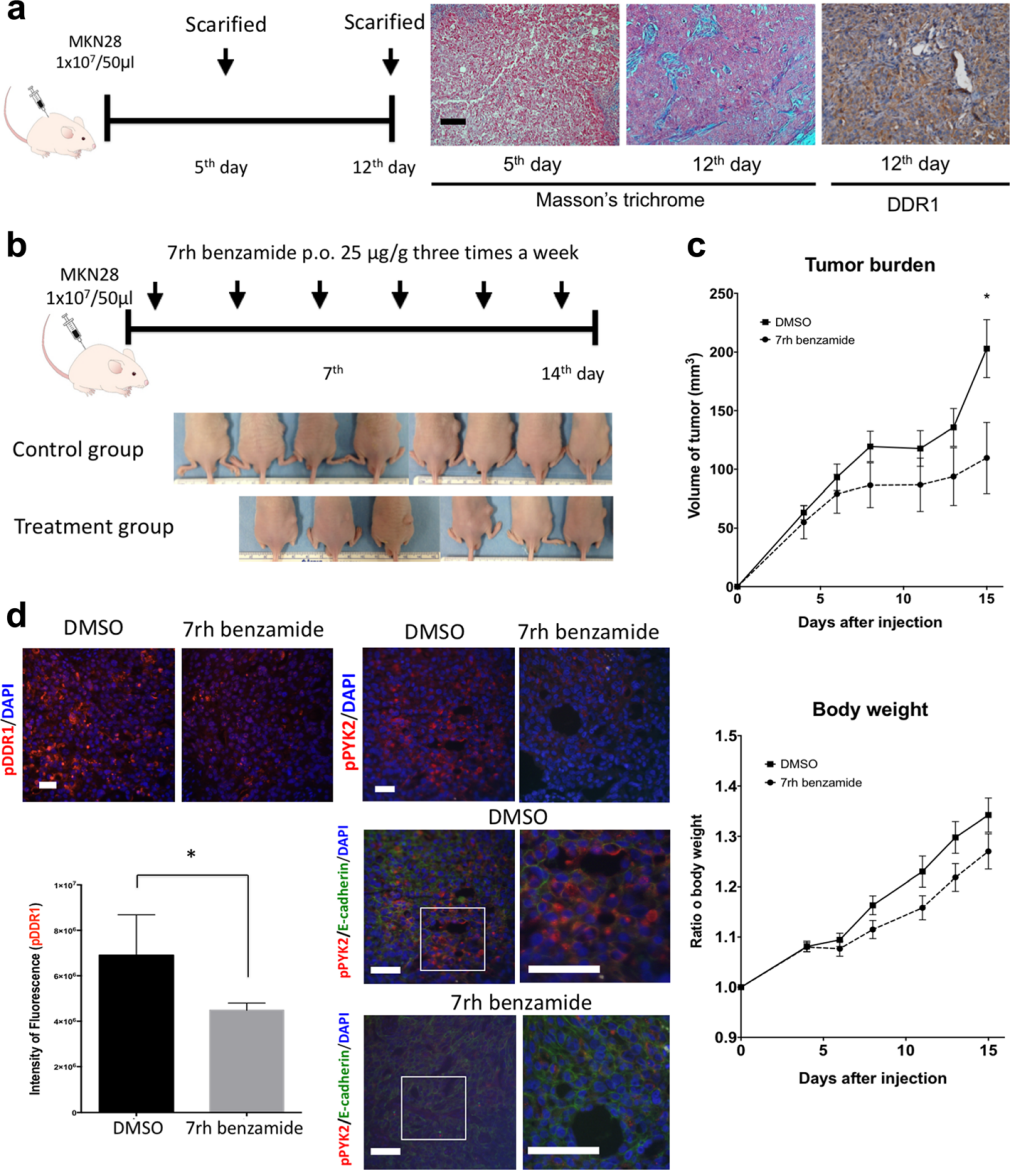
Pharmacologic inhibition of DDR1 reduces gastric cancer tumor growth. **a** MKN28 cells (1 × 10^7^ in 50 μl) were injected into the subcutaneous layer of athymic nude mice (*n* = 10). Five mice were sacrificed on day 5 post injection, and the remaining 5 mice were sacrificed after 12 days. Collagen in the tumor was assessed by Masson’s trichrome stain, and the expression of DDR1 was evaluated by immunohistochemistry. **b** MKN28 cells (1 × 10^7^ in 50 μl) were injected subcutaneously into nude mice. Animals were treated with vehicle (control; *n* = 8) or 25 μg/g of 7rh benzamide (7rh; *n* = 6) three times a week via oral gavage. The representative pictures of the mice right before sacrifice show the different tumor size between two groups. **c** Tumor volume and body weight were measured during drug administration, and tumors were harvested on day 17 post tumor cell injection. The error bars represent standard error of the mean (SEM) and an asterisk (*) represents *P* < 0.005, calculated by Mann-Whitney U test. **d** Harvested tumors were subjected to immunohistochemistry for phosphorylated DDR1 (pDDR1), phosphorylated PYK2 (pPYK2), and E-cadherin. Quantification of pDDR1 signal intensity is shown. Scale bar = 20 μm. The error bars represent standard error of the mean (SEM) and an asterisk (*) represents *P* < 0.005, calculated by Mann-Whitney U test

To investigate the effect of collagen-DDR1 signaling on tumor growth, the extent of collagen deposition in subcutaneous MKN28 gastric cancer xenografts was determined by histology using the Masson’s trichrome stain. Tumors harvested 5 or 12 days after tumor cell implantation displayed robust collagen accumulation, with the greatest collagen accumulation observed in tumors harvested on day 12. The level of DDR1 expression was also assessed by immunohistochemistry (Fig. 5a).

To determine if pharmacologic inhibition of DDR1 can affect tumor growth in vivo, a study using 7rh benzamide was conducted. Mice bearing subcutaneous MKN28 tumors were treated with 7rh benzamide or vehicle (Fig. 5b). DDR1 inhibition by 7rh benzamide significantly slowed tumor growth during the 2-week observation period but had no effect on body weight and animal activity (Fig. 5c).

Immunohistochemical analyses showed that tumors harvested from mice receiving short-term 7rh benzamide therapy had significantly reduced phosphorylated DDR1 and PYK2 levels when compared to tumors from control-treated mice. In tumors harvested from the control group, we observed that the expression of E-cadherin, a conventional epithelial cell marker, was decreased in regions with higher PYK2 phosphorylation. In tumors of experimental group, expression of E-cadherin was detected and PYK2 phosphorylation was significantly decreased in most part of tumor lesion (Fig. 5d).

## Discussion

In the current study, we demonstrated for the first time the correlation between DDR1 expression and worse overall prognosis in gastric cancer patients. Moreover, we identified that collagen-induced DDR1 activation enhanced aggressive tumor phenotypes, and that a pharmacologic inhibition of DDR1 can suppress cancer progression in vitro and in vivo. These observations provided strong evidence that DDR1 served as a critical mediator of gastric cancer aggressiveness and that the inhibition of DDR1 can be potentially used as a novel strategy to improve the prognosis of gastric cancer patients.

Prior to this study, the expression of DDR1 was unexplored in gastric cancers. However previous studies have demonstrated a positive association between DDR1 protein expression and poor prognosis in solid tumors, thus supporting the hypothesis that DDR1 expression enhances the aggressiveness of malignant tumors. Two studies reported that increased DDR1 expression was associated with poor survival of patients with non-small cell lung cancer [23, 28]. Elevated DDR1 expression in serous ovarian cancer was also indicated as a prognostic determinant [29]. A recent study reported that increased DDR1 expression was linked to a poor prognosis in pancreatic ductal adenocarcinoma [24]. Like other solid tumors, gastric cancers require the non-cancerous stromal portion of the primary tumors to enhance tumor growth and progression. In particular, several studies have described that tumor-associated stromal collagen can modulate immune responses within gastric cancers, and is associated with a poor prognosis in patients with advanced gastric cancers [30, 31]. Another report demonstrated that collagen could drive the migration of gastric cancer cells through the regulation of β-catenin [32]. Taken together, these findings supported the notion that the expression of DDR1, which uses extracellular collagen as a ligand, is a good candidate biomarker to predict gastric cancer prognosis.

In particular, through a multivariate analysis, we demonstrated that elevated DDR1 expression can be used as a biomarker for predicting poor survival in patients receiving adjuvant treatments after resection. In gastric cancers, adjuvant chemotherapy is generally prescribed for patients with advanced stage disease regardless of curative resection. In theory, circulating cancer cells from primary gastric cancers can metastasize into and colonize metastatic sites such as the peritoneum and liver after resection [33, 34]. Based on the results of the present study, a novel inhibitor of DDR1, 7rh benzamide, is potentially efficacious in preventing tumor recurrence after curative resection in DDR1-expressing gastric cancer through the suppression of cancer cell migration and tumorigenic abilities.

Several RTK inhibitors such as imatinib [14], nilotinib [13], and dasatinib [35] were shown to inhibit the DDR kinase activity. However, these agents displayed broad specificity. Recently Gao et al. described a novel and orally bioavailable specific inhibitor of DDR1, the 7rh compound of 3-(−2-(pyrazolo[1,5-a] pyrimidin-6-yl)-ethynyl) benzamides, which was shown to potently inhibit DDR1 activation in preclinical models [27]. The 7rh benzamide was synthesized using a palladium-catalyzed Sonogahira coupling, and showed a significant suppressive effect on the proliferation of DDR1-expressing cancer cells, but not DDR2-, Bcr-Abl-, or c-Kit-expressing cells. In our study, low concentrations of 7rh benzamide (0.18 or 0.54 μM) suppressed the activation of DDR1 and tumor cell activity by collagen in vitro. Moreover, in a xenograft model, the effect of 7rh benzamide to suppress tumor growth following subcutaneous injection of tumor cells was demonstrated. These findings indicated the potential use of 7rh benzamide as a novel agent to suppress gastric cancer progression.

DDR1 has been linked to invasion, migration, and survival of cells [22]. We observed that DDR1-induced PYK2 phosphorylation and loss of E-cadherin were induced by collagen stimulation, which were reduced upon DDR1 inhibition. In the xenograft model, we observed collagen accumulation as tumors increased in size. We also found that DDR1 inhibition reduced DDR1 activation, EMT, and tumor growth. Shintani et al. reported that type I collagen can trigger DDR1-induced PYK2 phosphorylation, and consequentially induced EMT through the downregulation of N-cadherin expression in pancreatic cancer cells [36]. EMT of cancer cells is associated with an aggressive phenotype and metastatic potential [37, 38]. We assessed the loss of the epithelial marker E-cadherin as a marker of EMT progression, because E-cadherin loss and/or mutation were shown to associate with progression and poor prognosis of gastric cancers [39, 40]. However, the correlation between DDR1 and E-cadherin expressions has remained elusive. Yeh et al. reported that DDR1 enhanced epithelial differentiation and promoted cell-to-cell junctions through E-cadherin stabilization in non-cancer cells [41]. Meanwhile, others reported that DDR1 upregulation promoted tumor progression by reducing E-cadherin expression in lung and colorectal cancers [42, 43]. The pro-survival effect of DDR1 in cancer cells also suggested its potential as a therapeutic target. The activation of Notch1 or the nuclear factor-kappaB pathway was suggested as the downstream signal that induces resistance to cytotoxic chemotherapy [44, 45]. However, the precise intracellular mechanism of DDR1-induced cancer cell aggressiveness is not clearly understood and requires further investigation.

## Conclusions

Our study demonstrated the potential of DDR1 as a new prognostic marker of gastric cancers. In particular, DDR1 expression was associated with poor survival in patients receiving adjuvant chemotherapy after resection for gastric cancers. We therefore suggest the use of specific DDR1 inhibitors as therapeutic agents to suppress gastric cancer progression.

## Additional files

Additional file 1: Figure S1. Validation of DDR1 antibody. An oligonucleotide targeting human DDR1 (5′-ACACUAAUAUAUGGACCUAGCUUGA-3′; siDDR1) was purchased from Integrated DNA Technologies (Coralville, IA). MKN45 cells in 100 μL of medium were seeded into 24 well plates and transfected with 750 ng of siDDR1 or control siRNA. DDR1 expression in siDDR1- and control siRNA-transfected cells was assessed by Western blot. (TIF 1917 kb)
Additional file 2: Figure S2. Relative ratio of cell viability. **A)** KATO-III and **B)** MKN28 cells were plated on collagen coated or non-collagen coated dishes and cell number determined on the days indicated by MTS assay. **C)** The effect of 7rh benzamide on KATO-III and **D)** MKN28 cell viability was determined by MTS assay. Cells were plated on non-coating or collagen coating dishes. The concentration of 7rh benzamide was 0, 0.06, 0.18, 0.54, 1.62 and 4.86 μM. (TIF 13503 kb)

## Abbreviations

DDR1: Discoidin domain receptor 1
DDRs: Discoidin domain receptors
EMT: Epithelial-mesenchymal transition
H&E: Hematoxylin and eosin
IHC: Immunohistochemistry
RTKs: Receptor tyrosine kinases
TMA: Tissue microarray

## References

[1] Ferlay J, Soerjomataram I, Dikshit R, Eser S, Mathers C, Rebelo M, Parkin DM, Forman D, Bray F (2015). Cancer incidence and mortality worldwide: sources, methods and major patterns in GLOBOCAN 2012. Int J Cancer.

[2] Bertuccio P, Chatenoud L, Levi F, Praud D, Ferlay J, Negri E, Malvezzi M, La Vecchia C (2009). Recent patterns in gastric cancer: a global overview. Int J Cancer.

[3] Bang YJ, Kim YW, Yang HK, Chung HC, Park YK, Lee KH, Lee KW, Kim YH, Noh SI, Cho JY (2012). Adjuvant capecitabine and oxaliplatin for gastric cancer after D2 gastrectomy (CLASSIC): a phase 3 open-label, randomised controlled trial. Lancet.

[4] Sakuramoto S, Sasako M, Yamaguchi T, Kinoshita T, Fujii M, Nashimoto A, Furukawa H, Nakajima T, Ohashi Y, Imamura H (2007). Adjuvant chemotherapy for gastric cancer with S-1, an oral fluoropyrimidine. N Engl J Med.

[5] Koizumi W, Narahara H, Hara T, Takagane A, Akiya T, Takagi M, Miyashita K, Nishizaki T, Kobayashi O, Takiyama W (2008). S-1 plus cisplatin versus S-1 alone for first-line treatment of advanced gastric cancer (SPIRITS trial): a phase III trial. Lancet Oncol.

[6] Wagner AD, Grothe W, Haerting J, Kleber G, Grothey A, Fleig WE (2006). Chemotherapy in advanced gastric cancer: a systematic review and meta-analysis based on aggregate data. J Clin Oncol.

[7] Wagner AD, Unverzagt S, Grothe W, Kleber G, Grothey A, Haerting J, Fleig WE (2010). Chemotherapy for advanced gastric cancer. Cochrane Database Syst Rev.

[8] Joseph SO, Wu J, Muggia FM (2012). Targeted therapy: its status and promise in selected solid tumors. Part II: impact on selected tumor subsets, and areas of evolving integration. Oncology (Williston Park).

[9] Li J, Chen F, Cona MM, Feng Y, Himmelreich U, Oyen R, Verbruggen A, Ni Y (2012). A review on various targeted anticancer therapies. Target Oncol.

[10] Cho JY (2013). Molecular diagnosis for personalized target therapy in gastric cancer. J Gastric Cancer.

[11] Fuchs CS, Tomasek J, Yong CJ, Dumitru F, Passalacqua R, Goswami C, Safran H, dos Santos LV, Aprile G, Ferry DR (2014). Ramucirumab monotherapy for previously treated advanced gastric or gastro-oesophageal junction adenocarcinoma (REGARD): an international, randomised, multicentre, placebo-controlled, phase 3 trial. Lancet.

[12] Bang YJ, Van Cutsem E, Feyereislova A, Chung HC, Shen L, Sawaki A, Lordick F, Ohtsu A, Omuro Y, Satoh T (2010). Trastuzumab in combination with chemotherapy versus chemotherapy alone for treatment of HER2-positive advanced gastric or gastro-oesophageal junction cancer (ToGA): a phase 3, open-label, randomised controlled trial. Lancet.

[13] Alves F, Vogel W, Mossie K, Millauer B, Hofler H, Ullrich A (1995). Distinct structural characteristics of discoidin I subfamily receptor tyrosine kinases and complementary expression in human cancer. Oncogene.

[14] Johnson JD, Edman JC, Rutter WJ (1993). A receptor tyrosine kinase found in breast carcinoma cells has an extracellular discoidin I-like domain. Proc Natl Acad Sci U S A.

[15] Vogel W, Gish GD, Alves F, Pawson T (1997). The discoidin domain receptor tyrosine kinases are activated by collagen. Mol Cell.

[16] Shrivastava A, Radziejewski C, Campbell E, Kovac L, McGlynn M, Ryan TE, Davis S, Goldfarb MP, Glass DJ, Lemke G (1997). An orphan receptor tyrosine kinase family whose members serve as nonintegrin collagen receptors. Mol Cell.

[17] Vogel W (1999). Discoidin domain receptors: structural relations and functional implications. FASEB J.

[18] Camara J, Jarai G (2010). Epithelial-mesenchymal transition in primary human bronchial epithelial cells is Smad-dependent and enhanced by fibronectin and TNF-alpha. Fibrogenesis Tissue Repair.

[19] Suh HN, Han HJ (2011). Collagen I regulates the self-renewal of mouse embryonic stem cells through alpha2beta1 integrin- and DDR1-dependent Bmi-1. J Cell Physiol.

[20] Wang CZ, Hsu YM, Tang MJ (2005). Function of discoidin domain receptor I in HGF-induced branching tubulogenesis of MDCK cells in collagen gel. J Cell Physiol.

[21] Yeh YC, Wang CZ, Tang MJ (2009). Discoidin domain receptor 1 activation suppresses alpha2beta1 integrin-dependent cell spreading through inhibition of Cdc42 activity. J Cell Physiol.

[22] Valiathan RR, Marco M, Leitinger B, Kleer CG, Fridman R (2012). Discoidin domain receptor tyrosine kinases: new players in cancer progression. Cancer Metastasis Rev.

[23] Ford CE, Lau SK, Zhu CQ, Andersson T, Tsao MS, Vogel WF (2007). Expression and mutation analysis of the discoidin domain receptors 1 and 2 in non-small cell lung carcinoma. Br J Cancer.

[24] Huo Y, Yang M, Liu W, Yang J, Fu X, Liu D, Li J, Zhang J, Hua R, Sun Y (2015). High expression of DDR1 is associated with the poor prognosis in Chinese patients with pancreatic ductal adenocarcinoma. J Exp Clin Cancer Res.

[25] Yamaguchi H, Sakai R (2015). Direct interaction between carcinoma cells and cancer associated fibroblasts for the regulation of cancer invasion. Cancers (Basel).

[26] Yashiro M, Hirakawa K (2010). Cancer-stromal interactions in scirrhous gastric carcinoma. Cancer Microenviron.

[27] Gao M, Duan L, Luo J, Zhang L, Lu X, Zhang Y, Zhang Z, Tu Z, Xu Y, Ren X (2013). Discovery and optimization of 3-(2-(Pyrazolo[1,5-a]pyrimidin-6-yl)ethynyl)benzamides as novel selective and orally bioavailable discoidin domain receptor 1 (DDR1) inhibitors. J Med Chem.

[28] Yang SH, Baek HA, Lee HJ, Park HS, Jang KY, Kang MJ, Lee DG, Lee YC, Moon WS, Chung MJ (2010). Discoidin domain receptor 1 is associated with poor prognosis of non-small cell lung carcinomas. Oncol Rep.

[29] Quan J, Yahata T, Adachi S, Yoshihara K, Tanaka K (2011). Identification of receptor tyrosine kinase, discoidin domain receptor 1 (DDR1), as a potential biomarker for serous ovarian cancer. Int J Mol Sci.

[30] Ohno S, Tachibana M, Fujii T, Ueda S, Kubota H, Nagasue N (2002). Role of stromal collagen in immunomodulation and prognosis of advanced gastric carcinoma. Int J Cancer.

[31] Nie XC, Wang JP, Zhu W, Xu XY, Xing YN, Yu M, Liu YP, Takano Y, Zheng HC (2013). COL4A3 expression correlates with pathogenesis, pathologic behaviors, and prognosis of gastric carcinomas. Hum Pathol.

[32] Li A, Zhou T, Guo L, Si J (2010). Collagen type I regulates beta-catenin tyrosine phosphorylation and nuclear translocation to promote migration and proliferation of gastric carcinoma cells. Oncol Rep.

[33] Mimori K, Fukagawa T, Kosaka Y, Kita Y, Ishikawa K, Etoh T, Iinuma H, Sasako M, Mori M (2008). Hematogenous metastasis in gastric cancer requires isolated tumor cells and expression of vascular endothelial growth factor receptor-1. Clin Cancer Res.

[34] Wu CH, Lin SR, Hsieh JS, Chen FM, Lu CY, Yu FJ, Cheng TL, Huang TJ, Huang SY, Wang JY (2006). Molecular detection of disseminated tumor cells in the peripheral blood of patients with gastric cancer: evaluation of their prognostic significance. Dis Markers.

[35] Day E, Waters B, Spiegel K, Alnadaf T, Manley PW, Buchdunger E, Walker C, Jarai G (2008). Inhibition of collagen-induced discoidin domain receptor 1 and 2 activation by imatinib, nilotinib and dasatinib. Eur J Pharmacol.

[36] Shintani Y, Fukumoto Y, Chaika N, Svoboda R, Wheelock MJ, Johnson KR (2008). Collagen I-mediated up-regulation of N-cadherin requires cooperative signals from integrins and discoidin domain receptor 1. J Cell Biol.

[37] Lamouille S, Xu J, Derynck R (2014). Molecular mechanisms of epithelial-mesenchymal transition. Nat Rev Mol Cell Biol.

[38] Steinestel K, Eder S, Schrader AJ, Steinestel J (2014). Clinical significance of epithelial-mesenchymal transition. Clin Transl Med.

[39] Corso G, Carvalho J, Marrelli D, Vindigni C, Carvalho B, Seruca R, Roviello F, Oliveira C (2013). Somatic mutations and deletions of the E-cadherin gene predict poor survival of patients with gastric cancer. J Clin Oncol.

[40] Xing X, Tang YB, Yuan G, Wang Y, Wang J, Yang Y, Chen M (2013). The prognostic value of E-cadherin in gastric cancer: a meta-analysis. Int J Cancer.

[41] Yeh YC, Wu CC, Wang YK, Tang MJ (2011). DDR1 triggers epithelial cell differentiation by promoting cell adhesion through stabilization of E-cadherin. Mol Biol Cell.

[42] Hu Y, Liu J, Jiang B, Chen J, Fu Z, Bai F, Jiang J, Tang Z (2014). MiR-199a-5p loss up-regulated DDR1 aggravated colorectal cancer by activating epithelial-to-mesenchymal transition related signaling. Dig Dis Sci.

[43] Miao L, Zhu S, Wang Y, Li Y, Ding J, Dai J, Cai H, Zhang D, Song Y (2013). Discoidin domain receptor 1 is associated with poor prognosis of non-small cell lung cancer and promotes cell invasion via epithelial-to-mesenchymal transition. Med Oncol.

[44] Das S, Ongusaha PP, Yang YS, Park JM, Aaronson SA, Lee SW (2006). Discoidin domain receptor 1 receptor tyrosine kinase induces cyclooxygenase-2 and promotes chemoresistance through nuclear factor-kappaB pathway activation. Cancer Res.

[45] Kim HG, Hwang SY, Aaronson SA, Mandinova A, Lee SW (2011). DDR1 receptor tyrosine kinase promotes prosurvival pathway through Notch1 activation. J Biol Chem.

