# Supplementary figures and images for: Discoidin domain receptor 1 activity drives an aggressive phenotype in gastric carcinoma
Source: BMC Cancer. 2017 Jan 31;17:87. doi: 10.1186/s12885-017-3051-9 (PMC5286810; doi:10.1186/s12885-017-3051-9)

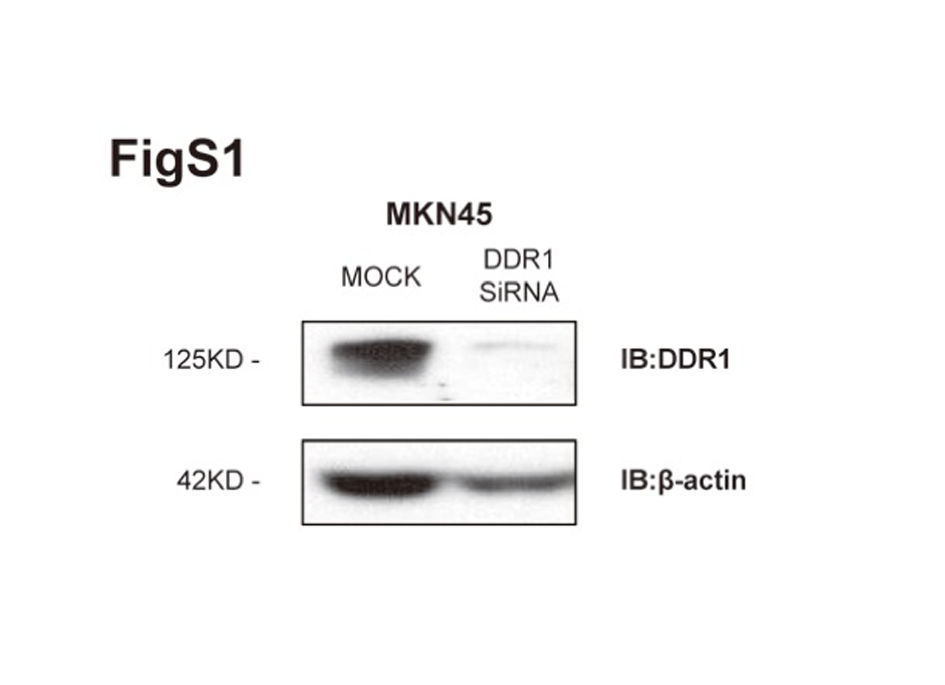

Supplement: Additional file 1: Figure S1. — Validation of DDR1 antibody. An oligonucleotide targeting human DDR1 (5′-ACACUAAUAUAUGGACCUAGCUUGA-3′; siDDR1) was purchased from Integrated DNA Technologies (Coralville, IA). MKN45 cells in 100 μL of medium were seeded into 24 well plates and transfected with 750 ng of siDDR1 or control siRNA. DDR1 expression in siDDR1- and control siRNA-transfected cells was assessed by Western blot. (TIF 1917 kb) [file 12885_2017_3051_MOESM1_ESM.tif]

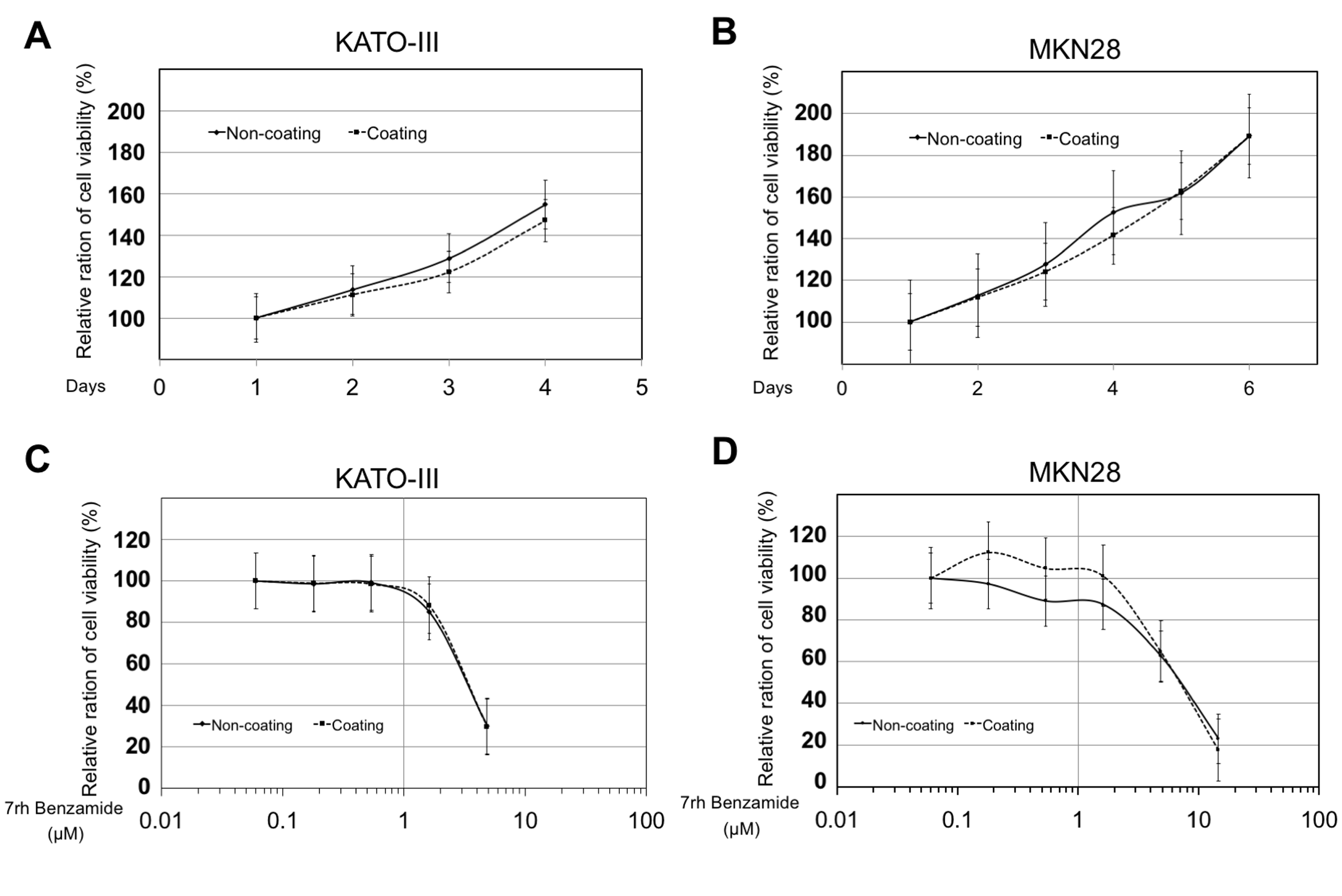

Supplement: Additional file 2: Figure S2. — Relative ratio of cell viability. A) KATO-III and B) MKN28 cells were plated on collagen coated or non-collagen coated dishes and cell number determined on the days indicated by MTS assay. C) The effect of 7rh benzamide on KATO-III and D) MKN28 cell viability was determined by MTS assay. Cells were plated on non-coating or collagen coating dishes. The concentration of 7rh benzamide was 0, 0.06, 0.18, 0.54, 1.62 and 4.86 μM. (TIF 13503 kb) [file 12885_2017_3051_MOESM2_ESM.tif]
